# Study on the Interaction Mechanism Between Soybean Protein Isolates and Lemon Flavor: Isomerization and Degradation of Citral

**DOI:** 10.3389/fnut.2022.929023

**Published:** 2022-07-21

**Authors:** Jun Guo, Jicheng Xu, Jie Chen

**Affiliations:** ^1^College of Biological and Food Engineering, Anhui Polytechnic University, Wuhu, China; ^2^Department of Chemistry and Material Engineering, Chizhou University, Chizhou, China; ^3^State Key Laboratory of Food Science and Technology, Jiangnan University, Wuxi, China

**Keywords:** soy protein isolate, citral, methyl heptenone, acetaldehyde, degradation

## Abstract

By headspace solid-phase microextraction/gas chromatography–mass spectrometry, the effects of 1% (w/v) alcohol denatured soybean protein isolates (L-SPI), native soybean protein isolates (N-SPI), as well as the thermal denaturation of soybean protein isolates (H-SPI) on low concentration (24 μmol/L) of citral was studied in aqueous. The results shows that the SPI could catalyze citral isomerization and yield methyl heptenone and acetaldehyde by inverse aldol condensation degradation. 3-Hydroxycitronelloal was formed as an intermediate in this reaction. The catalytic efficiency of the L-SPI was higher than that of N-SPI, whereas the catalytic efficiency of H-SPI was the lowest. Additionally, it shows that the catalytic efficiency increased as the pH increased. The catalytic efficiency of 7S (Soybean β-Conglycinin) was greater than that of 11S (Soy bean Proglycinin).

## Introduction

Plant protein beverage made from soybean protein isolate is cholesterol-free and has high amino acid content, resulting that plant protein beverage possess high nutritional value (1–3). However, due to the structure and properties of SPI, the flavor of vegetable beverage containing SPI is easily unbalanced (3–5). For example, when lemon juice is mixed with SPI, flavor changes occur. According to the literature (6), it is speculated that the functional groups of protein may had catalytic degradation of citral.

Citral was used in the preparation of strawberry, apple, apricot, sweet orange, and lemon flavors (7). Commercial citral usually typically contains 60% geranial and 40% neral, which are isomers (8). Geranial has a mild citrusy smell, whereas neral has a pungent grass smell (9). Lemongrass oil, lemon oil, and white lemon oil contain 70–80% citral (10). Citral can also be obtained from industrial geraniol (and nerol) by dehydrogenation using a copper catalyst. Moreover, it can be synthesized from dehydrolinalool using vanadium as a catalyst. Citral is used to manufacture citrus-based food flavors; However, as it is susceptible to oxidation, polymerization, and discoloration, it is usually used in foods that have neutral pH (11, 12); Citral is also used to synthesize iso-menthol and hydroxycitronella aldehyde and violanone, which is the raw material of vitamin A. Degradation of citral will lead to loss of the lemon-like aroma and produce off-flavor (10, 11, 13).

Citral is a terpenoid and the main flavor component of citrus foods (4, 14). Citral is an unsaturated aldehyde containing an isolated C = C double bond, C = O group, and C = C double bond conjugated with the carbonyl group that is easily degraded by other chemical components in food (15). For example, Wolken et al. (6) found that glycine and bovine serum albumin could catalyze the degradation of citral. The degradation of citral results in the loss of its unique aroma and the formation of other undesirable aroma components that lead to unpleasant alterations in the flavor of food (8).

This study to further explore the effects of SPI structures (Extracted from soybean meal) on the degradation of citral by inverse hydroxyl aldehyde condensation, and provide a theoretical reference for the flavor change in Lemon juice-SPI beverages.

## Materials and Methods

### Materials

Soybean (Suyun 626) was purchased from Fengyuan Seed Co. (Lian Yungang City, China). Citral (98%), acetaldehyde (98%), methyl Heptenone (98%) were provided by J&K Chemical Ltd. (Shanghai, China). 3-hydroxycitronellal (95%) was prepared as method of Fkyerat and Tabacchi (16). Neral (containing 10% geranial and 90% neral) and geranial (containing 95% geranial and 5% neral) were prepared by separating commercial citral by Wolken et al. (6). All other chemicals (analytical grade) were purchased from Sinopharm Chemical Reagent Company (Shanghai, China). Deionized water was used in all the experiments.

### Preparation of Protein Sample

#### Native-Soybean Protein Isolates

Native SPI was made from soybeans, by Jiang et al. (17) description, according to the alkaline pH extraction–isoelectric precipitation method. By the micro-Kjeldahl method, after solution of neutralization to pH 7.0, the protein content of SPI suspension was determined, by oven drying method (105°C overnight) and the total solid was determined. By the nitrogen conversion factor of 5.71 (18), the SPI extract was 92.1% by the calculated protein purity (dry weight basis). In deionized water, the 8% (w/v) SPI suspension was centrifuged, to remove particulates, at 10,000 × g, for 20 min. In the SPI suspension, the ionic strength, was 0.03–0.04 M, expressed as the concentration of NaCl which was determined using an S30 Seven Easy conductivity meter (Mettler Toledo GmbH Analytical, Sonnenberg strausse, Switzerland).

#### L-Soybean Protein Isolates

Native SPI usually has a beany smell, while use SPI as a food ingredient, it need to use ethanol to wash the defatted soybean meal, in order to remove the beany smell molecules of SPI. Broken after the soybean meal skim, skim for alcohol after wash, wash bad SPI flavor compounds in addition to further. followed by alcohol washing with anhydrous ethanol with solid/liquid ratio of 1:3 (w/v), stirring for 2 h, ethanol extraction and filtration to remove the ethanol, and alcohol washing for 2 times.

#### H-Soybean Protein Isolates

Preparation of fully heat-denatured protein (H-SPI, 1%, w/v): pre-heat denatured SPI at 100°C (preheat for 5 min first, and keep it for 20 min after reaching the set temperature).

#### 7S, 11S

The soy 11S and 7S protein fractions were isolated from soy flour by using the method of Sorgentini et al. (19).

### Preparation of Solutions

A stock solution containing 1,000 mg/L of citral was made up in methyl alcohol through gradient dilution, and made to give a SPI of 1% (w/v) solution of 20 mmol/L sodium phosphate/NaOH buffer, an aliquot of 24 μmol/L flavor of each 5 mL aliquots in 15 mL glass vials (AiXin Ltd., BeiJing. China). A separate solution containing a reference standard flavor was made up in a similar way. Three replications were made. The Solutions of the individual were used with small stirring bars at 37°C for 0.5 h to mixing them with the aroma compound in triplicates.

### Headspace Solid-Phase Microextraction Gas Chromatography-Mass Spectrometry (SPME-GC-MS) Analysis

The SPME holder for manual sampling and the SPME fibers, 50/30 μm polydimethylsiloxane (PDMS), were purchased from AnPu (ShangHai, China). The fibers were conditioned in the gas chromatograph injector port before use at the time and temperature recommended by the manufacturer. During the development of the headspace SPME method, the following parameters were optimized: type and thickness of fiber coating; headspace extraction time text; and sample agitation during extraction. For the aroma standards and the protein-flavor solutions, 5 mL aliquots were transferred into 15 mL glass vials (AiXin Ltd., BeiJing. China). The standards and the samples containing individual SPI were prepared in triplicates. KMO-2 basic magnetic stir bar (KeYi Ltd., GuangZhou, China) was placed in each sample vial. The samples were stirred at 250 min^–1^ and keeping the temperature constant at 37°C during the SPME extraction using an RW 20 magnetic stirring plate (KeYi Ltd., GuangZhou, China) under the water bath. After equilibration, the SPME fiber was exposed into the headspace of the sample vial for 30 min and was subsequently introduced into the gas chromatograph injector port for quantification.

Gas Chromatography. A Bluker SCION SQ456 GC/MS (Bruker, Kyoto, Japan) was used throughout the study. The column used was a Supelcowax 10 fused silica capillary column, 30 m, 0.25 mm inner diameter, 0.25 μm film thickness (Agilent DB-WAX). The carrier gas was helium (linear velocity; 0.8 mL/min). The injector port (splitless mode) temperature and the detector temperature were 250°C. The oven temperature was held isothermally at 120°C. Once the SPME sampling was completed, the fiber was immediately inserted into the gas chromatograph injector for desorption. The fiber was left in the port for 5 min for purging. There was no carry-over between samples using 7 min desorption time. Prior to the next SPME extraction, the fiber was allowed to cool to room temperature. The temperature was programmed, column temperature keeping at 40°C for 3 min, by heating the sample at a rate of 5°C/min to 90°C for the first phase and sample at a rate of 10°C/min to 230°C for the second phase, this temperature for 7 min (20).

A mass spectrometer was used to confirm the identity of volatile flavor compounds and further determine the potential volatile flavor byproducts that have been generated. The EI source for the mass spectrometer described above was operated at 70 eV.

### Qualitative and Quantitative Analysis Method of the Volatile Compounds

An external standard calibration was used to calculate the extent of binding. A stock solution containing 10 mmol/L of each flavor compound was created, using propylene glycol through gradient dilution and to an SPI of 1% (w/v) solution of 20 mmol/L phosphate buffer in 18 mL hermetically closed flasks (Kebeter, Beijing, China), stock solutions contained cital in each flask with or without SPI (blanks). Four replicates were created and shaken for 24 h at 37°C for equilibration.


$$\left[\text{L}\right]=\frac{{\left[H\,S\right]}_{P}}{{\left[H\,S\right]}_{C}}\times O$$

The values of [L] is the concentration of flavors in headspace, O is the flavor concentration (μmol/L) of control, [*HS*]*_*C*_* is the flavor compounds GC peak area of control, [*HS*]*_*P*_* is the flavor compounds GC peak area of sample.

### Measurement of Protein Fluorescence

Fluorescence intensity was measured using a Hitachi F-2700 Fluorescence Spectrophotometer (Hitachi Ltd., Tokyo, Japan). EX WL: 280.0 nm, EX Slit: 5.0 nm, EM Slit: 5.0 nm. Stock solutions of SPI (0.02% w/v) were prepared, 10 mmol/L citric acid phosphate buffer at pH 5–10. Flavors were added to solutions of SPI by diluting, respectively, propylene glycol at the concentration of 0–0.8 mmol/L. All the samples were prepared in plastic test tubes covered with aluminum foil. And the Solutions of the individual were used with small stirring bars at 37°C for 2 h to mixing them with the Aroma Compounds, in triplicates. stored at 4°C until use.

### Determination of Surface Hydrophobicity of Soybean Protein Isolates

By an 8-anilinonaphthalene-1-sulfonic acid (ANS) fluorescent probe method, in the aqueous solution, SPI hydrophobicity was determined with modifications (21). SPI was centrifuged at 12,000 × g, times is 15 min, temperature is 37°C, the supernatants were diluted in 20 mmol/L citric acid phosphate buffer (pH 5.0–pH9.0), obtain SPI concentrations ranging from 0.1 to 0.002 mg/mL. Subsequently, 20 μL of ANS (8.0 mmol/L in 0.1 M phosphate buffer, pH 7.2) was added to 2 mL of the diluted SPI solutions. Fluorescence intensity was measured using a Hitachi F-2700 Fluorescence Spectrophotometer (Hitachi Ltd., Tokyo, Japan) at 365 nm (excitation wavelength) and 520 nm (emission wavelength). The slope of the fluorescence intensity vs. protein concentration was used as an index of surface hydrophobicity (S0).

### Protein Solubility

The protein solubility of the SPI solutions was obtained by the method of Sorgentini et al. (19). SPI solutions were centrifuged at 12,000 × g for 15 min. The protein concentration of the supernatant was determined by Lowry (22). The protein solubility was calculated as the percentage of the protein concentration in the supernatant over that of the original SPI solution.

### Statistical Analysis

Differences between treatments were determined by analysis of variance (ANOVA) and Duncan’s Multiple Range test (*p* < 0.05) using statistical package SPSS 17.0 (SPSS Inc., Chicago, IL). Data were expressed as means ± standard deviations (SD) of triplicate determinations unless specifically mentioned.

## Results and Discussion

### Soybean Protein Isolates-Catalyzed Isomerization of Geranial and Neral

**FIGURE 1:**
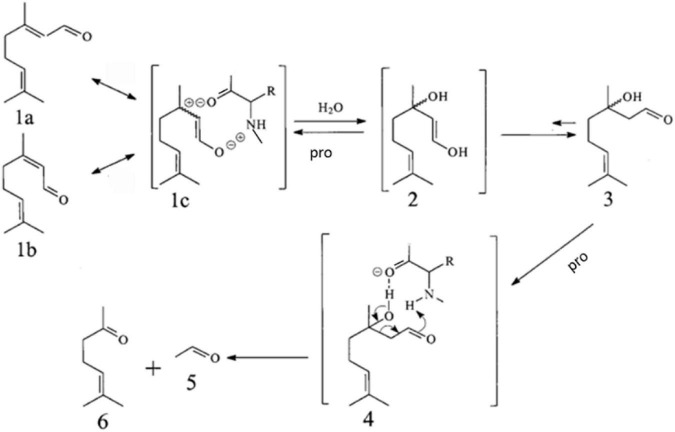
Proposed pathway for the isomerization of geranial (1a) and neral (1b) and their conversion *via* 3-hydroxycitronellal (3) into methyl heptenone (6) and acetaldehyde (5), catalyzed by Soy Protein Isolate (SPI), in alkaline aqueous solution.

**FIGURE 2:**
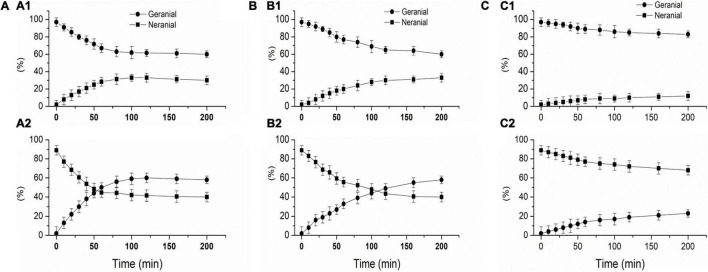
protein-catalyzed isomerization of geranial and neral, SPI 1%, 20 mmol/L potassium phosphate buffer (pH 7.0), Geranial and neral are expressed as a percentage of total citral. **(A)** L-SPI, **(B)** N-SPI, **(C)** H-SPI.

As seen in Figure 1, proteins and amino acid can catalyze the reverse hydroxyl aldehyde condensation reaction of citral to give methyl heptenone and acetaldehyde, with 3-hydroxycitronelloal as an intermediate (23–25). Moreover, as shown in Figure 2, SPI can catalyze the isomerization of geranial and neral, the two isomers of citral. Citral contains unsaturated conjugated double bonds. In the presence of polar amino acids, it is easily degraded by inverse hydroxyl aldehyde condensation to methyl heptenone and acetaldehyde.

According to the report, by X-ray diffraction method, Adachi et al. (26), Baud et al. (27), Maruyama et al. (28), and Maruyama et al. (29) determined that 7S subunit was connected to trimer through disulfide bond, and 11S peptide chain (A1aB1b, A2B1a, A1bB2, A3B4, and A5A4B3) was connected to trimer through disulfide bond, hexamer of 11S globulin is formed in face to face form by trimers. These trimers are hollow tubular structures with hydrophobic binding within or between trimers. Peptide chains of SPI contain amino acid side chains.

It is speculated that soybean protein can also catalyze the reverse hydroxyl aldehyde condensation degradation reaction of citral to produce methyl heptenone and acetaldehyde.

Figure 2 shows that SPI can catalyze the isomerization of geranial and neral. Figure 2A1 shows that L-SPI catalyze the isomerization of geranial into neral, Figure 2A2 shows that L- SPI catalyze the isomerization of neral into geranial. Figure 2B1 shows that N-SPI catalyze the isomerization of geranial into neral, Figure 2B2 shows that N-SPI catalyze the isomerization of neral into geranial. Figure 2C1 shows that H-SPI catalyze the isomerization of geranial into neral, Figure 2C2 shows that H-SPI catalyze the isomerization of neral into geranial.

It can be seen in Figures 2A1,2 that the use of L-SPI led to the most rapid isomerization of citral. After 100 min, the L-SPI was found to isomerize 37% of pure geranial to neral, and 60% of pure neral to geranial. It can be seen in Figures 2B1,B2 that N-SPI has a lower efficiency in catalyzing citral isomerization, as the reaction reaches equilibrium only after 200 min. H-SPI catalyzed the isomerization of citral with the lowest efficiency (Figures 2C1,C2).

It has been reported (6) that glycine and bovine serum albumin can catalyze the isomerization of geranial or neral under neutral reaction conditions. In addition, Kuwahara et al. (30) have reported the enzyme-catalyzed isomerization of citral. They found that the reaction was balanced with 40% of the mixture being neral and 60% as geranial. Wolken et al. (6) have reported the isomerization of geranyl and neral at room temperature (25°C) using glycine or bovine serum albumin. Kimura et al. (31) found that geranial can also be isomerized in acidic solutions to form neral. Therefore, it can be concluded that while SPI is mixed with citral, SPI maybe catalyze the isomerization of geranial or neral.

### Protein—Catalyzed Citral to Methyl Heptenone

**FIGURE 3:**
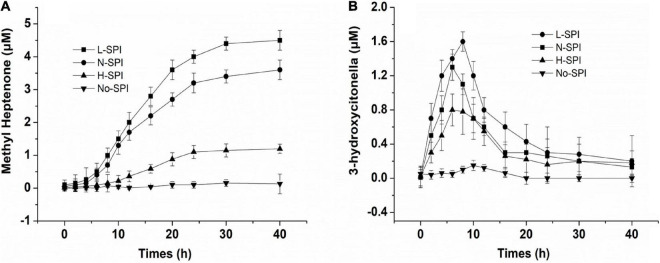
Effect of different treated protein on the SPI—catalyzed to citral **(A,B)**, the concentration of protein is 1% (w/v), 20 mmol/L potassium phosphate buffer, citral’s primary concentration is 24 μmol/L.

Citral, methyl heptanone, and 3-hydroxy-citronelloal can be detected in the sample after citral and SPI exist in the aqueous solution. Figure 3 shows the catalytic efficiency of alcohol-washed SPI (L-SPI), native SPI (N-SPI), and high-temperature denatured SPI (H-SPI) on citral degradation. Of these, L-SPI was the most effective catalyst, followed by N-SPI and H-SPI. Methyl heptenone was not formed when only the buffer was used.

**FIGURE 4:**
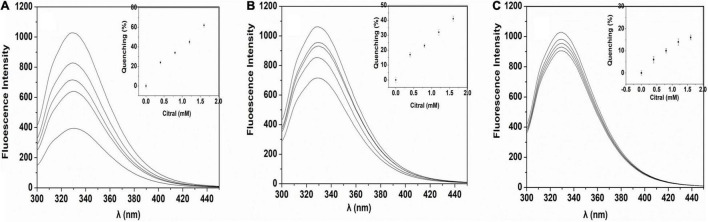
Fluorescence of SPI in citral aqueous solution. **(A)** L-SPI, **(B)** N-SPI, **(C)** H-SPI, the concentration of protein is 0.02% (w/v), 20 mM potassium phosphate buffer and, and citral’s primary concentration is 0.4–1.6 mmol/L.

The oil and water-soluble carbohydrate moieties of the SPI were removed after alcohol treatment, and more binding sites of SPI were exposed. From Figure 4A, Maximum fluorescence quenching intensity was observed for L-SPI. Form the Table 1, L-SPI has the best solubility and the lowest surface hydrophobicity between L-SPI, N-SPI and H-SPI. It has been reported that the process of alcohol washing to obtain L-SPI results in a certain degree of SPI denaturation, thereby changing the protein conformation and altering the nature of the flavor compounds (32), The SPI structure becomes more orderly after alcohol treatment. This finding conformed to the report by Tanford, who reported the characteristic denaturation of the protein structure by organic solvents (33).

**TABLE 1:** Surface hydrophobicity and protein solubility of N-SPI, H-SPI, L-SPI.

	N-SPI	H-SPI	L-SPI
Solubility (%)	83^b^ ± 4	63^c^ ± 6	96^a^ ± 4
Surface hydrophobicity (S_0_)	214^b^ ± 8	365^a^ ± 12	198^c^ ± 10

*Values are means and standard deviations of three determinations, the different lower case letters (a–c) in the same row indicate significant difference among the values at the p < 0.05.*

Treatment with alcohol weakens the hydrophobic forces, but strengthens the hydrogen bonding and electrostatic forces, thereby ensuring that the hydrophobic core is not damaged. Alcohol-washing treatment damages the outer structure of the soy protein and its loose and disorderly hydrophilic outer structure is transformed into the β-spiral structure. This conformation is conducive to the formation of hydrogen bonds, which results in a high degree of hydration and the protein molecules assuming a highly spiral state. Denaturation after the alcohol-washing treatment imparts more freedom to the hydrophilic chain segments in the SPI molecules, increasing its ability to move rapidly. Thus, this finding suggests that alcohol-washed soybean protein can result in the flexibility of SPI, effectively increasing its solubility (34).

According to the literature (35), It speculated that the maximum binding force was observed for the interaction of L-SPI and citral, and the most polar hydrophilic groups between L-SPI, N-SPI and H-SPI. Therefore, L-SPI had a higher probability of binding to citral and catalyzing its conversion to methyl heptenone.

N-SPI is a natural protein that exists in a metastable state. The crosslinked network structure of the protein is flexible and comprises a hydrophilic peptide chain (36). Citral molecules could move into the internal spaces and bind to the polar fragment of the protein, which led to its conversion into methyl heptenone and acetaldehyde. From Figure 4B, Fluorescence quenching was observed for the mixture of N-SPI and citral. Form the Table 1, N-SPI has the more solubility and the more surface hydrophobicity than H-SPI. According to the literature (35, 37), the binding of N-SPI and citral is a possibility; thus, N-SPI could bind citral and catalyze citral to methyl heptenone. It maybe that SPI is an enzyme with conformational adaptability and participates in the reorganization of the polypeptide segments at the binding sites.

From Figure 4C, Minimum fluorescence quenching intensity was observed for H-SPI. Form the Table 1, H-SPI have the worst solubility and the highest surface hydrophobicity between L-SPI, N-SPI, and H-SPI. Thermal denaturation resulted in H-SPI losing its metastable state. The space formed within by the three-dimensional reticular structure collapsed, leading to the unfolding of the advanced structure (18). Subsequently, the orderly and compact structure of SPI was converted into an undefined peptide structure, resulting in a loss of biological and catalytic activity (1). The molecular surface structure also exhibited changes; the hydrophilic groups were relatively reduced. Moreover, the groups that were originally hidden within several groups of hydrophobic molecules were exposed to the molecular surface, resulting in the protein particles not mixing with water and losing their water film. Additionally, it was easy for the entangled molecules to collide with each other, which further resulted in the destruction of the binding sites that were likely responsible for catalytic activity. The aggregation phenomenon caused dissociation and heat accumulation in the protein solution. Heat treatment of proteins in the folding state results in a state of instability because the surface hydrophobicity is enhanced when the protein hydrophobic core is exposed. Next, the internal space of the inner hydrophilic chain participates in the catalytic reaction. The interaction of citral molecules with SPI shows that the catalytic reaction may be difficult owing to the strong surface area hydrophobicity. Thus, as the citral molecule is not within the binding pocket of the protein, the rate of the catalytic reaction is very slow. It can be inferred from the SPI catalytic reaction that citral molecules enter the sphere of the soybean protein, the catalytic site may be located on the SPI hydrophilic polar group.

### Effect of pH on the Protein-Catalyzed Conversion of Citral to Methyl Heptenone

**FIGURE 5:**
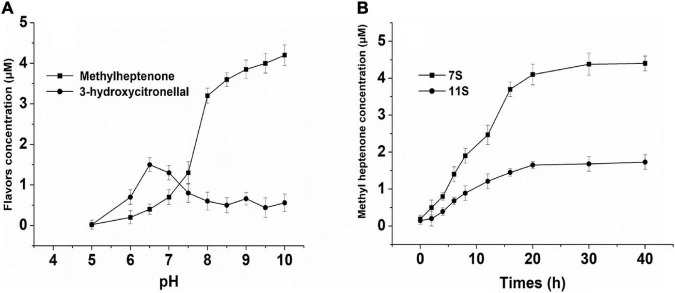
**(A)** Effect of different pH solution of NSPI—catalyzed to citral. **(B)** Effect of 7S, 11S-catalyzed to citral. The concentration of protein is 1% (w/v), 20 mmol/L potassium phosphate buffer/NaOH, and primary citral’s concentration is 24 μmol/L.

In this study, we found that SPI could catalyze the degradation of citral by reverse condensation; the end product was methyl heptenone and the intermediate was 3-hydroxycitronelloal (Figure 5A). No products were formed at pH 5. As the pH was increased from 5 to 7.5, the yield of 3-hydroxycitronelloal was found to be greater than that of methyl heptenone. As the pH was further increased from 7.5 to 10, the methyl heptanone yield was found to be higher than that of 3-hydroxyl citronelloal.

The study of the degradation reactions of citral mainly include those that are acid catalyzed. Kimura et al. (11) used NMR and IR to study the deacetylation and degradation of citral into terpenes in an acidic environment. In addition, it has been reported that citral is easily oxidized and that compounds such as BHT and BHA have a protective antioxidant effect on citral (8, 12, 13), however, our findings were contradictory to those reported in the literature. We found that the reverse hydroxyl aldehyde condensation degradation reaction of citral is suitable in an alkaline environment. Our findings were consistent with the results of Wolken et al. (6) who reported that glycine and bovine serum albumin catalyze the reverse hydroxyl aldehyde condensation degradation reaction of citral in neutral or dilute alkali solution to yield methyl heptenone and acetaldehyde.

### 7S and 11S—Catalyzed Citral to Methyl Heptenone

The effects of 7S and 11S on the SPI-catalyzed conversion of citral are shown in Figure 5B. The rate of conversion of citral by 7S was higher than that achieved by 11S. The deacetylation of citral by SPI is likely attributed to the enzyme and substrate combination, both of which can change the structure of mutual induction, eventually forming a suitable complex and ensuring the completion of the catalytic process (38).

Due to the large number of hydrophobic groups on the surface of the basic subunit of 11S, spontaneous aggregation is easy to occur in the solution, so the solubility is lower in the range of pH 4.5∼8.0 (39, 40). This phenomenon may cause the 11S protein to mask part of the active site. 11S has a compact globular structure, low solubility, and low molecular flexibility when mixed with citral (41, 42). 7S is a trimer, the dissociated trimer will have more flexible mobile regions than the 11S hexamer, it maybe have more active site (26). The levels of the hydrophilic and polar amino acids in 7S are higher than in 11S (28). So 7S-catalyzed rate is also high. Based on the above analysis, we found that 7S had higher catalytic efficiency than 11S.

## Conclusion

The results shows that the SPI could catalyze citral isomerization and yield methyl heptenone and acetaldehyde by reaction of inverse aldol condensation degradation. 3-Hydroxycitronelloal was formed as an intermediate in this reaction. The catalytic efficiency of the L-SPI was higher than that of N-SPI, whereas the catalytic efficiency of H-SPI was the lowest of L-SPI, N-SPI and H-SPI. Additionally, we found that the catalytic efficiency increased as the pH increased. The catalytic efficiency of 7S was greater than that of 11S. The catalytic site may be located on the SPI hydrophilic polar group. From this study it can be known that the reaction of SPI catalyze citral isomerization and yield methyl heptenone and acetaldehyde could change the flavor of Citrus juice-SPI beverages.

## Data Availability Statement

The original contributions presented in this study are included in the article/supplementary material, further inquiries can be directed to the corresponding author/s.

## Author Contributions

JG: writing the manuscript. JX: participate in experimental design and guidance. JC: guidance and others offer the materials. All authors contributed to the article and approved the submitted version.

## Conflict of Interest

The authors declare that the research was conducted in the absence of any commercial or financial relationships that could be construed as a potential conflict of interest.

## Publisher’s Note

All claims expressed in this article are solely those of the authors and do not necessarily represent those of their affiliated organizations, or those of the publisher, the editors and the reviewers. Any product that may be evaluated in this article, or claim that may be made by its manufacturer, is not guaranteed or endorsed by the publisher.
